# Anti-Allergic and Anti-Inflammatory Effects of Kuwanon G and Morusin on MC/9 Mast Cells and HaCaT Keratinocytes

**DOI:** 10.3390/molecules24020265

**Published:** 2019-01-11

**Authors:** Seong Eun Jin, Hyekyung Ha, Hyeun-Kyoo Shin, Chang-Seob Seo

**Affiliations:** Herbal Medicine Research Division, Korea Institute of Oriental Medicine, 1672 Yuseong-daero, Yuseong-gu, Daejeon 34054, Korea; noellajin@kiom.re.kr (S.E.J.); hkha@kiom.re.kr (H.H.); hkshin@kiom.re.kr (H.-K.S.)

**Keywords:** Kuwanon G, morusin, anti-allergy, anti-skin inflammation

## Abstract

Atopic dermatitis (AD) is a prevalent chronic inflammatory skin disease. The use of immunomodulatory corticosteroids in AD treatment causes adverse side effects. Therefore, novel natural anti-inflammatory therapeutics are needed. The aim of the present study was to investigate the anti-allergic and anti-inflammatory activities of kuwanon G and morusin. To investigate the effect of kuwanon G and morusin on skin inflammation, enzyme-linked immunosorbent assays (ELISA) to quantitate secreted (RANTES/CCL5), thymus- and activation-regulated chemokine (TARC/CCL17), and macrophage-derived chemokine (MDC/CCL22) were performed, followed by Western blotting to measure the phosphorylation of signal transducer and activator of transcription 1 (STAT1) and nuclear transcription factor-κB (NF-κB) p65 in tumor necrosis factor-α (TNF-α) and interferon-γ (IFN-γ)-stimulated HaCaT keratinocytes. In order to evaluate the anti-allergic effects, ELISA to quantify histamine and leukotriene C_4_ (LTC_4_) production and Western blotting to measure 5-lipoxygenase (5-LO) activation were performed using PMA and A23187-stimulated MC/9 mast cells. Kuwanon G reduced the release of RANTES/CCL5, TARC/CCL17, and MDC/CCL22 via down-regulation of STAT1 and NF-κB p65 signaling in TNF-α and IFN-γ-stimulated HaCaT keratinocytes. Kuwanon G also inhibited histamine production and 5-LO activation in PMA and A23187-stimulated MC/9 mast cells. Morusin inhibited RANTES/CCL5 and TARC/CCL17 secretion via the suppression of STAT1 and NF-κB p65 phosphorylation in TNF-α and IFN-γ-stimulated HaCaT keratinocytes, and the release of histamine and LTC_4_ by suppressing 5-LO activation in PMA and A23187-stimulated MC/9 mast cells. Kuwanon G and morusin are potential anti-inflammatory mediators for the treatment of allergic and inflammatory skin diseases such as AD.

## 1. Introduction

Atopic dermatitis (AD) is a multifunctional and heterogenous chronic inflammatory skin disease characterized by different clinical phenotypes based on its complex interactions between genetic and multiple environmental factors including chemical allergens [1,2]. It is characterized by the infiltration of T-helper (Th)-2 cells (Th2) and T-cell subsets, such as Th1, Th17, and Th22 cells, into skin lesions and the activation of several inflammatory cells, such as mast cells, keratinocytes, lymphocytes, macrophages, and eosinophils [3,4]. Chemokines are small proteins secreted by various cell types, including keratinocytes and immune cells, and the level of chemokines is closely correlated to the severity of AD [5,6].

Keratinocytes play a pivotal role in the pathogenesis of inflammatory skin diseases, such as AD [7]. Activated keratinocytes secrete Th2-related chemokines, including regulated on activation, normal T-cell expressed and secreted (RANTES/CCL5), thymus- and activation-regulated chemokine (TARC/CCL17), and macrophage-derived chemokine (MDC/CCL22). These Th2-related chemokines stimulate the infiltration of immune cells, including mast cells, monocytes, and T lymphocytes, into the site of inflammation on the skin and cause the inflammatory skin diseases [8,9,10]. Therefore, the downregulation of RANTES/CCL5, TARC/CCL17, and MDC/CCL22 in keratinocytes may be a potential target for treating inflammatory skin diseases [11,12].

Mast cells mediate various immune responses and regulate allergic inflammation, including in AD [13]. Activated mast cells secrete inflammatory mediators, such as histamine, leukotrienes, prostaglandin E_2_, and cytokines, thorough degranulation. These inflammatory mediators contribute to inflammation via the recruitment and activation of immune cells [14], and in particular, cause histamine-mediated pruritus in AD patients [15]. Thus, inhibitors of these inflammatory mediators can be used for the treatment of inflammatory and allergic diseases.

Commonly used medications to treat AD are anti-inflammatory corticosteroids; however, the long-term usage of these steroids has limitations, because they can cause skin atrophy and systemic adverse effects [16,17]. Therefore, the development of new natural candidates is required to treat and alleviate AD without any side effects.

Our previous report demonstrated that *Morus alba* L. inhibited the TARC/CCL17 release in tumor necrosis factor-α (TNF-α) and interferon-γ (IFN-γ)-stimulated HaCaT keratinocytes, and suppressed the development of atopic dermatitis-like lesions induced by the house dust mite in NC/Nga mice [18]. It is reported that morusin, one of the marker compounds contained in *M. alba* L., has an anti-tumorigenic effect in gastric [19], lung [20], hepatocellular [21], breast [22], and prostate cancer [23]. Kuwanon G, another marker compound contained in *M. alba* L., has been reported to have anti-atherosclerosis [24] and anti-asthma [25] effects, as well as anti-bacterial activity against oral pathogens [26]. However, the anti-allergic and anti-inflammatory effects of kuwanon G and morusin in keratinocytes and mast cells have not been clarified.

In the present study, we investigated whether kuwanon G and morusin inhibit the secretion of RANTES/CCL5, TARC/CCL17, and MDC/CCL22 in HaCaT keratinocytes and the release of histamine and leukotriene C_4_ (LTC_4_) in MC/9 mast cells. In addition, we studied the molecular mechanisms underlying the anti-allergic and anti-inflammatory actions of kuwanon G and morusin.

## 2. Results

### 2.1. High-Performance Liquid Chromatography (HPLC) Analysis of the Two Bioactive Marker Compounds in M. alba L.

**Figure 1:**
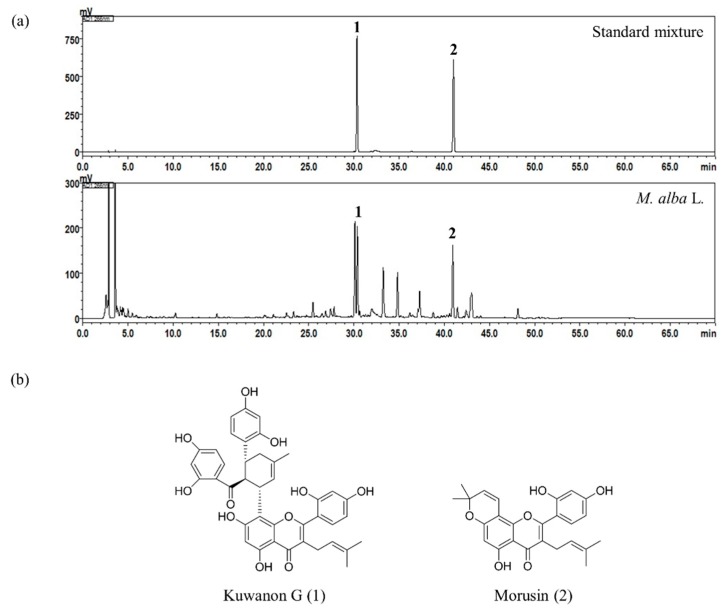
HPLC chromatograms of the standard mixture and *M. alba* L. sample at a UV detection wavelength of 266 nm (**a**) and chemical structures of the two bioactive marker compounds (**b**).

Using the established HPLC-photo-diode array (PDA) method, the two flavones, kuwanon G and morusin, in *M. alba* L. were simultaneously determined and eluted at 30.39 and 40.96 min, respectively. The HPLC-PDA chromatograms of standard mixture and 70% ethanol extract of the *M. alba* L. sample are shown in Figure 1a. Regression equations of the two bioactive compounds, kuwanon G and morusin, were y = 27,995.89x − 54,747.25 and y = 53,046.55x − 51,240.77, respectively, with a determination coefficient of 0.9998 at concentration ranges of 0.31–20.00 μg/mL and 1.56–100.00 μg/mL. The quantitation of kuwanon G and morusin was monitored at 266 nm. Based on the above results, the amounts of the two bioactive marker compounds, kuwanon G and morusin, in the *M. alba* L. root bark were found to be 2.26 ± 0.01 mg/g and 1.12 ± 0.01 mg/g with relative standard deviations of 0.47% and 0.83%, respectively.

### 2.2. Effects of Kuwanon G and Morusin on HaCaT and MC/9 Cell Viability

**Figure 2:**
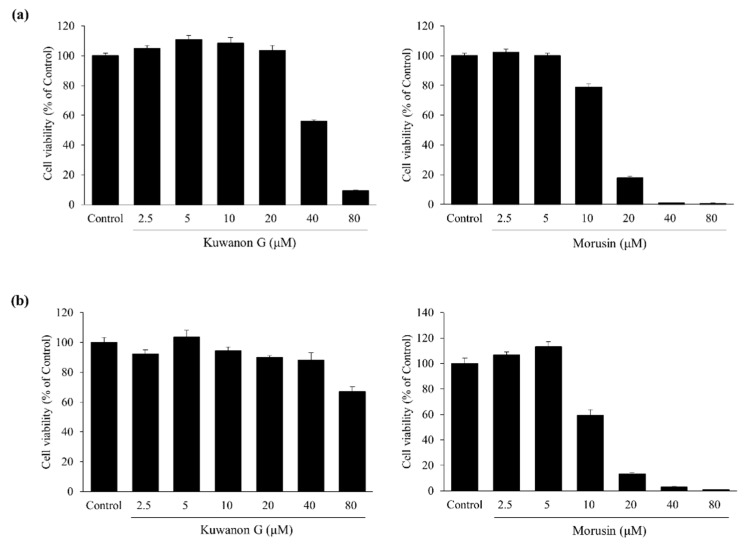
Cell viabilities of kuwanon G and morusin in HaCaT keratinocytes (**a**) and MC/9 mast cells (**b**). Data are expressed as the mean ± SEM (*n* = 4).

In HaCaT keratinocytes, kuwanon G and morusin did not alter the cell viability at concentrations up to 20 and 5 μM, respectively (Figure 2a). Kuwanon G and morusin did not affect MC/9 mast cell viability at concentrations up to 10 and 5 μM, respectively (Figure 2b). All subsequent experiments were conducted at nontoxic concentrations.

### 2.3. Effects of Kuwanon G and Morusin on the Chemokine Production in HaCaT Keratinocytes

**Figure 3:**
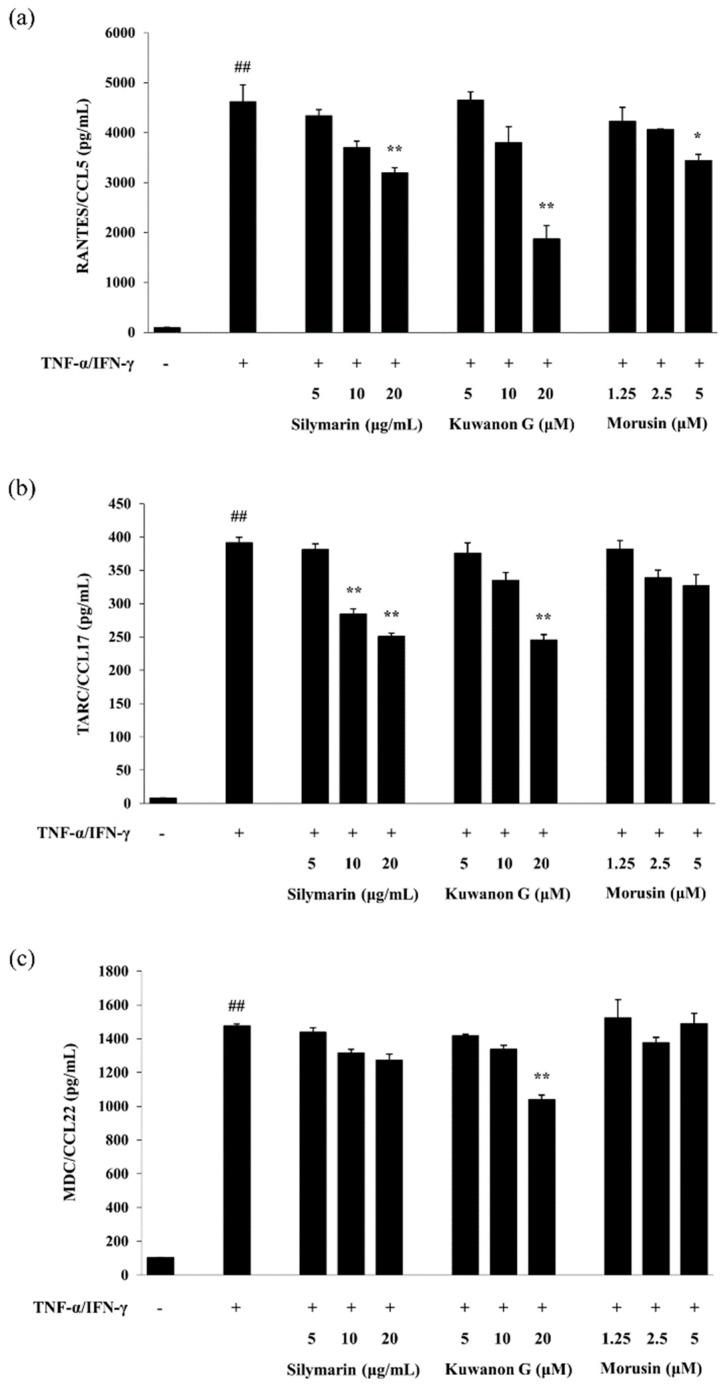
Effects of kuwanon G and morusin on the production of RANTES/CCL5 (**a**), TARC/CCL17 (**b**), and MDC/CCL22 (**c**) by TNF-α and IFN-γ-stimulated HaCaT keratinocytes. Silymarin was used as a positive control for the inhibition of chemokine production. Data are expressed as the mean ± SEM (*n* = 3). ^##^ *p* < 0.01 versus vehicle control cells; * *p* < 0.05 and ** *p* < 0.01 versus TNF-α and IFN-γ-treated cells.

As presented in Figure 3, treatment with TNF-α and IFN-γ significantly increased the level of RANTES/CCL5, TARC/CCL17, and MDC/CCL22 secreted by HaCaT keratinocytes compared with the vehicle control (*p* < 0.01). Kuwanon G at a high concentration significantly decreased TNF-α and IFN-γ-induced RANTES/CCL5, TARC/CCL17, and MDC/CCL22 production in HaCaT keratinocytes (*p* < 0.01). Morusin decreased the level of RANTES/CCL5 (*p* < 0.05) and TARC/CCL17 in a dose-dependent manner, but had no effect on the MDC/CCL22 production when compared with that of the TNF-α and IFN-γ-treated HaCaT keratinocytes.

### 2.4. Effects of Kuwanon G and Morusin on Signal Transducer and Activator of Transcription 1 (STAT1) and Nuclear Transcription Factor-κB (NF-κB) Phosphorylation in HaCaT Keratinocytes

**Figure 4:**
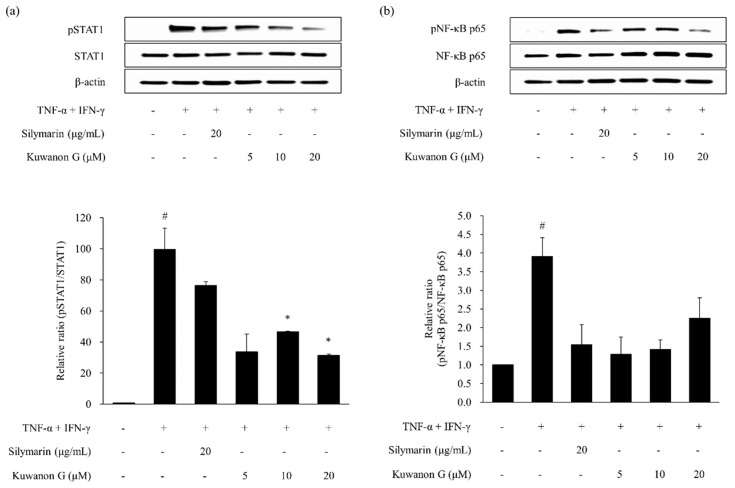
Effect of kuwanon G on TNF-α and IFN-γ-induced STAT1 (**a**) and NF-κB p65 (**b**) phosphorylation in HaCaT keratinocytes. Expression of total and phosphorylated STAT1 (pSTAT1) and NF-κB p65 (pNF-κB p65) was determined by Western blotting. Silymarin was used as the positive control. Data are expressed as the mean ± SEM (*n* = 2). *^#^ p* < 0.05 versus vehicle control cells; * *p* < 0.05 versus TNF-α and IFN-γ-treated cells.

**Figure 5:**
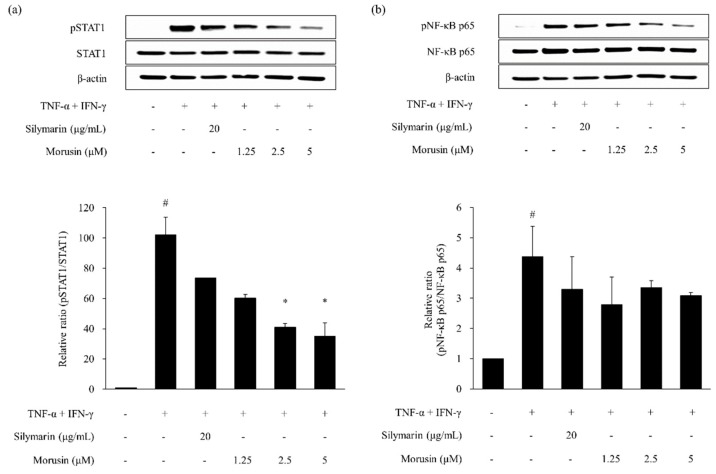
Effect of morusin on TNF-α and IFN-γ-induced STAT1 (**a**) and NF-κB p65 (**b**) phosphorylation in HaCaT keratinocytes. Expression of total and phosphorylated STAT1 (pSTAT1) and NF-κB p65 (pNF-κB p65) was determined by Western blotting. Silymarin was used as the positive control. Data are expressed as the mean ± SEM *(n* = 2). ^#^ *p* < 0.05 versus vehicle control cells; * *p* < 0.05 versus TNF-α and IFN-γ-treated cells.

As shown in Figure 4 and Figure 5, the phosphorylation of STAT1 and NF-κB p65 was increased by TNF-α and IFN-γ treatment in HaCaT keratinocytes (*p* < 0.05). The phosphorylation of STAT1 and NF-κB p65 was decreased by silymarin, which served as a positive control. Kuwanon G and morusin reduced the expression of phosphorylated STAT1 (pSTAT1) (*p* < 0.05) and NF-κB p65 (p NF-κB p65) in TNF-α and IFN-γ-stimulated HaCaT keratinocytes when compared to the HaCaT keratinocytes treated with TNF-α and IFN-γ alone.

### 2.5. Effects of Kuwanon G and Morusin on the Production of Histamine and LTC_4_ in MC/9 Mast Cells

**Figure 6:**
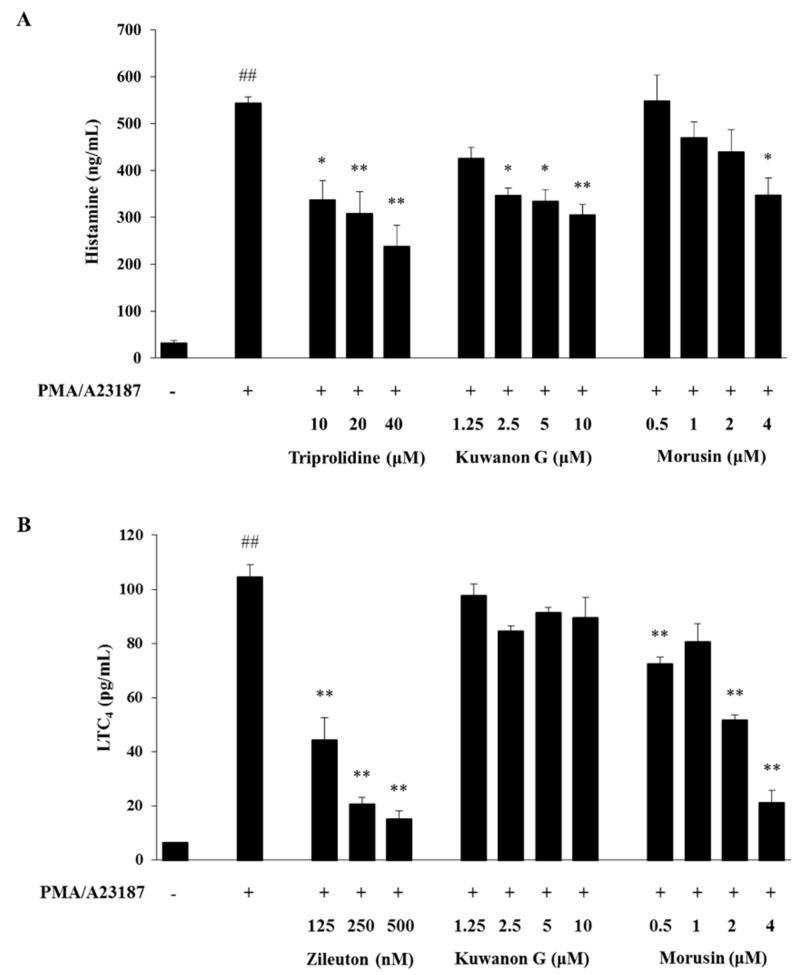
Effects of kuwanon G and morusin on the production of histamine (**a**) and LTC_4_ (**b**) in PMA and A23187-stimulated MC/9 mast cells. Triprolidine and zileuton were used as positive controls for the inhibition of histamine and LTC_4_ production, respectively. Data are expressed as the mean ± SEM (*n* = 3). ^##^ *p* < 0.01 versus vehicle control cells; * *p* < 0.05 and ** *p* < 0.01 versus PMA and A23187-treated cells. LTC_4_, leukotriene C_4_.

As shown in Figure 6, treatment with phorbol 12-myristate 13-acetate (PMA) and A23187 significantly increased the level of histamine and LTC_4_ in the culture supernatant of MC/9 mast cells compared with treatment with vehicle control (*p* < 0.01). Triprolidine and zileuton, which were used as positive controls, significantly reduced PMA and A23187-induced histamine and LTC_4_ production, respectively, when compared with the cells treated with PMA and A23187 alone (*p* < 0.01). Similarly, kuwanon G reduced the PMA and A23187-induced histamine production compared with the only PMA and A23187-treated cells (*p* < 0.01), but had no significant effect on the PMA and A23187-induced LTC_4_ production. On the other hand, morusin suppressed both histamine (*p* < 0.05) and LTC_4_ (*p* < 0.01) production in the PMA and A23187-treated cells.

### 2.6. Effects of Kuwanon G and Morusin on the 5-lipoxygenase (5-LO) Activation in MC/9 Mast Cells

**Figure 7:**
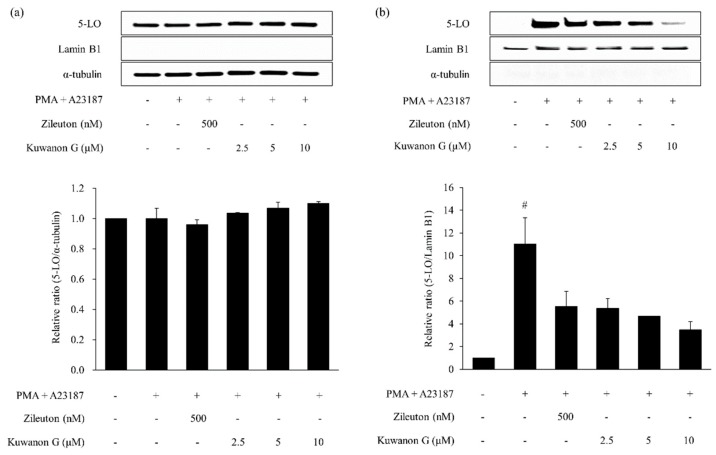
Effect of kuwanon G on PMA and A23187-induced 5-LO activation in MC/9 mast cells. Expression of cytoplasmic (**a**) and nuclear (**b**) 5-LO was determined by Western blotting. Zileuton was used as the positive control. Data are expressed as the mean ± SEM (*n* = 2). ^#^ *p* < 0.01 versus vehicle control cells.

**Figure 8:**
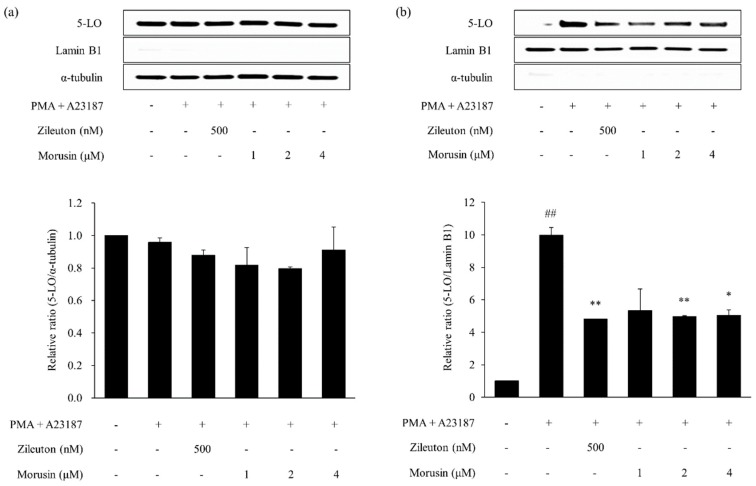
Effect of morusin on PMA and A23187-induce 5-LO activation in MC/9 mast cells. Expression of cytoplasmic (**a**) and nuclear (**b**) 5-LO was determined by Western blotting. Zileuton was used as the positive control. Data are expressed as the mean ± SEM (*n* = 2). ^##^ *p* < 0.01 versus vehicle control cells; * *p* < 0.05 and ** *p* < 0.01 versus PMA and A23187-treated cells.

Treatment with PMA and A23187 significantly increased nuclear 5-LO in MC/9 mast cells when compared with the vehicle-treated cells (*p* < 0.05, Figure 7 and Figure 8). In contrast, zileuton (positive control) significantly reduced the 5-LO protein expression in the nucleus (*p* < 0.01) of PMA and A23187-treated cells. Further, treatment with kuwanon G and morusin markedly decreased the PMA and A23187-induced 5-LO activation.

## 3. Discussion

Over the last 30 years, the prevalence of AD has increased. Although immunomodulatory drugs such as corticosteroids were used to treat AD, the risk of side effects by steroids has been reported. AD is one of the most common skin disorders seen in infants and children, and develops in the first year of life in 60% of affected individuals [27]. Although immunomodulatory drugs such as corticosteroids were used to treat AD, the risk of side effects by steroids has been reported, especially there remain issues such as possible adverse effects with chronic use in infants and children with severe AD. By contrast, the agents originated from natural products are commonly possessed high bioavailability and lower toxicity. However, unregulated flavonoid-containing supplements may have biological activities that can adverse effect to human health [28]. Further studies for closer examination of the bioavailability and absorption of kuwanon G and morusin are needed, however, it will have more benefits than corticosteroids when infants and children only take the proper amount of flavone-containing supplement. Thus, there is a need to develop effective and safer drugs for treatment of inflammatory skin diseases like AD [29]. This study evaluated the effect of kuwanon G and morusin on AD-like skin inflammation in HaCaT keratinocytes and MC/9 mast cells and characterized the possible mechanisms of action.

The stimulation of keratinocytes with TNF-α and IFN-γ induces the secretion of pro-inflammatory Th2 lymphocyte chemokines, which are involved in the infiltration of inflammatory cells into the site of inflammation on the skin [9,10]. Several studies reported that the level of TARC production was significantly higher when stimulated with both TNF-α and IFN-γ, compared with when treated with either TNF-α or IFN-γ alone [30,31]. Therefore, we used TNF-α and IFN-γ as activators of atopic dermatitis-like response in HaCaT keratinocytes. The secretion of inflammatory chemokines by keratinocytes is regulated by transcription factors via the STAT1 and NF-κB signaling pathway [9,10]. Several studies demonstrated that STAT1 and NF-κB play crucial roles in the chemokine production mediated by TNF-α and IFN-γ in keratinocytes [9,10,32]. Stimulation of keratinocytes with TNF-α and IFN-γ results in the activation of STAT1 and NF-κB signaling [10]. In this study, TNF-α and IFN-γ induced the production of RANTES/CCL5, TARC/CCL17, and MDC/CCL22, and activated STAT1 and NF-κB signaling pathway in HaCaT keratinocytes. In contrast, kuwanon G inhibited the release of Th2 lymphocyte chemokines via downregulation of RANTES/CCL5, TARC/CCL17, and MDC/CCL22 thorough inhibition of the STAT1 and NF-κB signaling pathway in TNF-α and IFN-γ-stimulated HaCaT keratinocytes. In addition, morusin suppressed the production of TNF-α and IFN-γ-induced RANTES/CCL5 and TARC/CCL17 via the down-regulation of STAT1 and NF-κB signaling in HaCaT keratinocytes. Similar to our study, several studies have reported the inhibitory effects of natural products against inflammatory skin diseases by targeting the signaling pathway leading to STAT1 and NF-κB activation [9,10,33,34,35].

The leukotrienes are lipid mediators synthesized from arachidonic acid (AA) by the action of the 5-LO, leukotriene A_4_ (LTA_4_) hydrolase and LTC_4_ synthase. Leukotrienes are secreted into the extracellular space and play a crucial role in the inflammatory response to injury [36]. After cellular stimulation, 5-LO is translocated to the nuclear membrane and activated by 5-LO-activating protein (FLAP), a nuclear membrane protein [37]. During this process, 5-LO catalyzes the oxygenation of AA, followed by a dehydration step to produce the epoxide intermediate LTA_4_. LTA_4_ is converted into leukotriene B_4_ or is conjugated with glutathione to form LTC_4_, the cysteinyl leukotriene compound. LTC_4_ secreted from mast cells stimulates of bronchoconstriction, mucous secretion, and vasodilation and increases the post-capillary venule permeability [38]. After the degranulation of mast cells, fever and an itching sensation are induced in the skin lesion by inflammatory mediators, such as histamine and LTC_4_ [39]. Severe itching and scratching behavior are aggravated during in AD [39,40]. Therefore, inhibitors of histamine, LTC_4_, or 5-LO have been used to alleviate the allergic symptoms. Mast cells are activated by protein kinase C (PKC), elevation of intracellular calcium levels, activation of phospholipase D (PLD), and tyrosine kinase, which promote the movement of granule including histamine, and fusion with the plasma membrane [41,42,43,44]. PMA and A23187 used for activating MC/9 mast cells in our study are highly potent PKC activator and calcium ionophore, respectively [44]. In several reports, PMA and A23187 treatment significantly elevated the histamine release in MC/9 mast cells [45,46]. Therefore, we used PMA and A23187 as activators for degranulation of MC/9 mast cells in this study.

Recently, Kim demonstrated the effects of *Costaria costata* extract on atopic dermatitis via suppressing expression of TARC, eotaxin, TNF-α, and IL-6 in TNF-α and IFN-γ-stimulated HaCaT keratinocytes as well as release histamine in PMA and A23187-stimulated MC/9 mast cells [45]. In our results, PMA and A23187 induced the activation of 5-LO and the production of histamine and LTC_4_ in MC/9 mast cells. We found that kuwanon G has the potential to alleviate allergic diseases via the inhibition of histamine release. Although kuwanon G did not significantly inhibit the LTC_4_ production, it markedly decreased 5-LO activation in PMA and A23187-stimulated MC/9 mast cells. Therefore, kuwanon G may have therapeutic potential against allergic diseases, including AD, via the suppression of histamine release and 5-LO-related pathway and the influence of kuwanon G on the release of histamine and LTC_4_ may differ due to the diversity in degranulation mechanisms. In addition, morusin inhibited the PMA and A23187-induced production of both histamine and LTC_4_ in MC/9 mast cells. Thus, we suggest that morusin might mitigate mast cell-mediated allergic reactions through the inhibition of histamine and LTC_4_. The inhibitory effect of morusin on the production of LTC_4_ can be attributed to the suppression of 5-LO activation.

## 4. Materials and Methods

### 4.1. Plant Materials

Dried root bark of *M. alba* L. (Moraceae) was purchased from Kwangmyungdang Medicinal Herbs (Ulsan, Republic of Korea) in June 2016 and the manufacture’s serial number is K1012201512. The origin of the sample was identified by Dr. Goya Choi, Herbal Medicine Research Division, Korea Institute of Oriental Medicine (Daejeon, Republic of Korea). The voucher specimen (2016-ST12-2) has been deposited at the Herbal Medicine Research Division, Korea Institute of Oriental Medicine.

### 4.2. Chemicals and Reagents

Morusin (purity: 99.8%, PubChem CID: 5281671) and kuwanon G (purity: 99.0%, PubChem CID: 5281667) were purchased from Biopurify Phytochemicals (Chengdu, China). The chemical structures of the two reference compounds are shown in Figure 1b. HPLC-grade solvents, methanol, acetonitrile, and water, were obtained from JT Baker (Phillipsburg, NJ, USA). ACS reagent, formic acid (for analysis, purity: ≥98%, PubChem CID: 284) was purchased from Merck KGaA (Darmstadt, Germany). Dulbecco’s modified Eagle’s medium (DMEM), fetal bovine serum (FBS), penicillin-streptomycin, phosphate-buffered saline (PBS) and NE-PER Nuclear and Cytoplasmic Extraction Reagents were purchased from Thermo Fisher Scientific (Waltham, MA, USA). T-Cell Culture Supplement with conA (T-STIM) and Cell Counting Kit-8 (CCK-8) were obtained from Becton, Dickinson & Co. (Franklin Lakes, NJ, USA) and Dojindo (Kumamoto, Japan), respectively. 2-Mercaptoethanol, PMA, A23187, triprolidine, zileuton, silymarin and CelLytic™ MT Cell Lysis Reagent were bought from Sigma-Aldrich (St. Louis, MO, USA). Human recombinant TNF-α, IFN-γ, and enzyme-linked immunosorbent assay (ELISA) kits for RANTES/CCL5, TARC/CCL17, and MDC/CCL22 were purchased from R&D Systems Inc. (Minneapolis, MN, USA). Histamine and LTC_4_ ELISA kits were obtained from Oxford Biomedical Research Inc. (MI, USA) and Cayman Chemical Co. (Ann Arbor, MI, USA), respectively. Protease inhibitor cocktail and Bio-Rad Protein Assay Reagent were obtained from Roche Applied Science (Indianapolis, IN, USA) and Bio-Rad Laboratories (Hercules, CA, USA), respectively. Polyvinylidene fluoride (PVDF) membrane was purchased from Millipore Corp. (Bedford, MA, USA). Specific antibodies against 5-LO, α-tubulin, pSTAT1, pNF-κB p65, and β-actin were bought from Cell Signaling Technology (Danvers, MA, USA). Primary antibodies against lamin B1 and STAT1 were obtained from Abcam (Cambridge, United Kingdom), and the antibody against NF-κB p65 was purchased from Santa Cruz Biotechnology (Dallas, TX, USA). Horseradish peroxidase (HRP)-conjugated secondary antibodies against mouse and rabbit immunoglobulins were bought from Jackson ImmunoResearch (West Grove, PA, USA). SuperSignal™ West Pico PLUS Chemiluminescent Substrate was obtained from Thermo Fisher Scientific (Rockford, IL, USA).

### 4.3. Preparation of 70% Ethanol Extract of M. alba L.

The dried *M. alba* L. root bark (10.0 kg) was extracted with 70% ethanol (100 L) for 3 h at 80 °C using an electric extractor, KSP-240L (Kyungseo Machine Co., Incheon, Republic of Korea). To eliminate the extract solvent, the extract solution was filtered using a standard sieve (No. 270, 53 μm; Chung Gye Sang Gong Sa, Seoul, Republic of Korea), concentrated using the EV-1020 Digital Rotary evaporator (SciLab Korea Co., Ltd., Seoul, Republic of Korea) under vacuum, and freeze-dried (Genesis 25LL, Virtis Co., Gardiner, NY, USA) to give a powdered sample. The amount of lyophilized 70% ethanol extract was 1.2 kg (12.0%).

### 4.4. HPLC Analysis of the Two Bioactive Marker Compounds in M. alba L.

The HPLC equipment used for the quantitative analysis of the two flavones, kuwanon G and morusin, in *M. alba* L. was a Shimadzu LC-20A series (Kyoto, Japan) consisting of a pump (LC-20AT), degassing unit (DGU-20A3R), column oven (CTO-20A), auto sampler (SIL-20A), and PDA detector (SPD-M20A). The acquisition and processing of chromatographic data was performed using the LabSolution software (Version 5.53, SP3, Shimadzu, Kyoto, Japan). The column used for the separation of kuwanon G and morusin was the Phenomenex Gemini C18 analytical column (250 mm × 4.6 mm, 5 μm, Torrance, CA, USA), which was maintained at 45 °C. The mobile phase for the efficient separation of analytes was composed of 0.1% (*v*/*v*) aqueous formic acid (A) and acetonitrile (B), and flowed from the initial 20% B to 90% B for 50 min. The flow rate was kept constant at 1.0 mL/min, the injection volume was 10 μL, and the PDA scanned for quantification was 190–400 nm. For HPLC analysis of the two biomarker compounds in *M. alba* L., 500.0 mg of the ground raw *M. alba* L. sample material was dissolved in 20 mL of 70% methanol and extracted for 60 min at 25°C using a Branson ultra-sonicator, 8510E-DTH (Danbury, CT, USA). Then, the extracted solution was filtered through a 0.2 μm membrane filter (Pall Life Sciences, MI, USA), before injection into the HPLC equipment.

### 4.5. Cell Culture

The human keratinocyte cell line, HaCaT, was obtained from CLS Cell Lines Service GmbH (Eppelheim, Baden-W¨urttemberg, Germany). HaCaT keratinocytes were cultured in DMEM supplemented with 10% heat-inactivated FBS, penicillin (100 U/mL), and streptomycin (100 μg/mL) in a 5% CO_2_ incubator at 37 °C. The murine mast cell line, MC/9, was obtained from the American Type Culture Collection (ATCC; Rockville, MD). MC/9 mast cells were cultured in DMEM supplemented with 10% heat-inactivated FBS, 10% T-STIM, 0.05 mM 2-mercaptoethanol, penicillin (100 U/mL), and streptomycin (100 μg/mL) in a 5% CO_2_ incubator at 37 °C. 

### 4.6. Cytotoxicity Assay

A cell viability assay was performed to determine the cytotoxicity of kuwanon G and morusin using the CCK-8 kit. HaCaT keratinocytes (2 × 10^3^ cells/well) and MC/9 mast cells (3 × 10^3^ cells/well) were incubated in 96-well plates with various concentrations of the compounds for 24 h. The CCK-8 reagent was added to each well, followed by incubation for 4 h. The absorbance (Abs) at 450 nm was measured using a Benchmark plus microplate reader (Bio-Rad Laboratories, Hercules, CA, USA). Cell viability was calculated using the following equation: cell viability (%) = (mean Abs in test sample wells/mean Abs in control wells) × 100. Data are expressed as the means ± standard error of mean (SEM; *n* = 4).

### 4.7. Measurement of Chemokine Levels

HaCaT keratinocytes (1 × 10^6^ cells/well) were cultured in 6-well plates. After confluence was reached, the cells were treated with various concentrations of the compounds in the absence or presence of TNF-α and IFN-γ (10 ng/mL each) for 24 h. The levels of RANTES/CCL5, TARC/CCL17, and MDC/CCL22 in the culture supernatants were determined using ELISA kits. Silymarin was used as a positive control for the chemokines. Data are expressed as the means ± SEM (*n* = 3).

### 4.8. Measurement of Histamine and Leukotriene C4 Levels

MC/9 mast cells (5 × 10^4^ cells/well) were incubated in 48-well plates with various concentrations of the compounds in the absence or presence of PMA (50 nM) and A23187 (1 μM) for 24 h. The levels of histamine and LTC_4_ in the culture supernatants were determined using ELISA kits. Triprolidine and zileuton were used as positive controls for histamine and LTC_4_, respectively. Data are expressed as the means ± SEM (*n* = 3).

### 4.9. Western Blotting

To evaluate the effect of kuwanon G and morusin on the expression of pSTAT1 and pNF-κB p65 in HaCaT keratinocytes, the cells were treated with various concentrations of the compounds in the absence of TNF-α and IFN-γ for 30 min. Whole-cell extract was prepared using a CelLytic™ MT Cell Lysis Reagent containing protease inhibitor cocktail. To determine the effect of kuwanon G and morusin on expression of 5-LO in MC/9 mast cells, the cells were treated with various concentrations of the compounds in the absence of PMA and A23187 for 1 h. Nuclear and cytoplasmic extracts were extracted using NE-PER Nuclear and Cytoplasmic Extraction Reagents according to the manufacturer’s protocol. The protein concentration was determined using a Bio-Rad Protein Assay Reagent. Equal amounts of proteins (15–20 μg/mL) were separated by 4–15% gradient sodium dodecyl sulfate-polyacrylamide gel electrophoresis (SDS-PAGE) and transferred onto a PVDF membrane. The membrane was blocked with 5% skim milk in Tris-buffered saline containing 1% Tween 20 (TBST), followed by overnight incubation at 4 °C with primary antibodies; 5-LO, lamin B1, α-tubulin, pSTAT1, STAT1, pNF-κB p65, NF-κB p65, and β-actin. The membrane was washed thrice with TBST and incubated with a corresponding HRP-conjugated secondary antibody for 1 h at 25 °C. The membrane was washed thrice with TBST and the bands visualized using a SuperSignal™ West Pico PLUS Chemiluminescent Substrate. Images were captured using Chemi-Doc (Bio-Rad Laboratories) and quantification was performed using the Image Lab software (Version 5.2, Bio-Rad Laboratories). Zileuton was used as a positive control for the inhibition of 5-LO activation. Silymarin was used as a positive control for pSTAT1 and NF-κB p65 inhibition. Data are expressed as the means ± SEM (*n* = 2).

### 4.10. Statistical Analysis

The data were expressed as the mean ± SEM and analyzed using one-way analysis of variance and Bonferroni multiple comparisons test (SYSTET, Version 10.0, Systat Software Inc., San Jose, CA, USA). To analyze the variance for the changes in protein expression, *t*-test was performed. *p* < 0.05 was considered statistically significant.

## 5. Conclusions

In conclusion, we found that kuwanon G and morusin inhibited chemokine production by blocking of STAT1 and NF-κB pathways in keratinocytes, and reduced the release of histamine and LTC4 by suppressing the 5-LO activation in MC/9 mast cells. Our results suggest that both kuwanon G and morusin may be useful as anti-inflammatory mediators for the prevention or treatment of AD-like skin inflammatory conditions and other allergic diseases based on their inhibitory effects on chemokine production. To better elucidate the exact roles of kuwanon G and morusin, further studies are required in animal models, such as AD.

## References

[1] Lee K.S., Chun S.Y., Lee M.G., Kim S., Jang T.J., Nam K.S. (2018). The prevention of TNF-α/IFN-γ mixture-induced inflammation in human keratinocyte and atopic dermatitis-like skin lesions in Nc/Nga mice by mineral-balanced deep sea water. Biomed. Pharmacother..

[2] D’Auria E., Banderali G., Barberi S., Gualandri L., Pietra B., Riva E., Cerri A. (2016). Atopic dermatitis: Recent insight on pathogenesis and novel therapeutic target. Asian Pac. J. Allergy Immunol..

[3] Bieber T. (2008). Atopic dermatitis. N. Engl. J. Med..

[4] Weidinger S., Novak N. (2016). Atopic dermatitis. Lancet.

[5] Pease J.E., Williams T.J. (2006). Chemokines and their receptors in allergic disease. J. Allergy Clin. Immunol..

[6] Jahnz-Rozyk K., Targowski T., Paluchowska E., Owczarek W., Kucharczyk A. (2005). Serum thymus and activation-regulated chemokine, macrophage-derived chemokine and eotaxin as markers of severity of atopic dermatitis. Allergy.

[7] Jung M.R., Lee T.H., Bang M.H., Kim H., Son Y., Chung D.K., Kim J. (2012). Suppression of thymus- and activation-regulated chemokine (TARC/CCL17) production by 3-O-β-D-glucopyanosylspinasterol via blocking NF-κB and STAT1 signaling pathways in TNF-α and IFN-γ-induced HaCaT keratinocytes. Biochem. Biophys. Res. Commun..

[8] Bernard F.X., Morel F., Camus M., Pedretti N., Barrault C., Garnier J., Lecron J.C. (2012). Keratinocytes under Fire of Proinflammatory Cytokines: Bona Fide Innate Immune Cells Involved in the Physiopathology of Chronic Atopic Dermatitis and Psoriasis. J. Allergy (Cairo).

[9] Park J.H., Kim M.S., Jeong G.S., Yoon J. (2015). Xanthii fructus extract inhibits TNF-α/IFN-γ-induced Th2-chemokines production via blockade of NF-κB, STAT1 and p38-MAPK activation in human epidermal keratinocytes. J. Ethnopharmacol..

[10] Ju S.M., Song H.Y., Lee S.J., Seo W.Y., Sin D.H., Goh A.R., Kang Y.H., Kang I.J., Won M.H., Yi J.S. (2009). Suppression of thymus- and activation-regulated chemokine (TARC/CCL17) production by 1,2,3,4,6-penta-O-galloyl-beta-D-glucose via blockade of NF-kappaB and STAT1 activation in the HaCaT cells. Biochem. Biophys. Res. Commun..

[11] Kwon D.J., Bae Y.S., Ju S.M., Goh A.R., Youn G.S., Choi S.Y., Park J. (2012). Casuarinin suppresses TARC/CCL17 and MDC/CCL22 production via blockade of NF-κB and STAT1 activation in HaCaT cells. Biochem. Biophys. Res. Commun..

[12] Yano C., Saeki H., Komine M., Kagami S., Tsunemi Y., Ohtsuki M., Nakagawa H. (2015). Mechanism of Macrophage-Derived Chemokine/CCL22 Production by HaCaT Keratinocytes. Ann. Dermatol..

[13] Barrett N.A., Austen K.F. (2009). Innate cells and T helper 2 cell immunity in airway inflammation. Immunity.

[14] Kawakami T., Ando T., Kimura M., Wilson B.S., Kawakami Y. (2009). Mast cells in atopic dermatitis. Curr. Opin. Immunol..

[15] Ikoma A., Rukwied R., Ständer S., Steinhoff M., Miyachi Y., Schmelz M. (2003). Neuronal sensitization for histamine-induced itch in lesional skin of patients with atopic dermatitis. Arch. Dermatol..

[16] Carr W.W. (2013). Topical calcineurin inhibitors for atopic dermatitis: Review and treatment recommendations. Paediatr. Drugs.

[17] Flohr C., Irvine A.D. (2013). Systemic therapies for severe atopic dermatitis in children and adults. J. Allergy Clin. Immunol..

[18] Lim H.S., Ha H., Lee H., Lee J.K., Lee M.Y., Shin H.K. (2014). Morus alba L. suppresses the development of atopic dermatitis induced by the house dust mite in NC/Nga mice. BMC Complement. Altern. Med..

[19] Wang F., Zhang D., Mao J., Ke X.X., Zhang R., Yin C., Gao N., Cui H. (2017). Morusin inhibits cell proliferation and tumor growth by down-regulating c-Myc in human gastric cancer. Oncotarget.

[20] Yin X.L., Lv Y., Wang S., Zhang Y.Q. (2018). Morusin suppresses A549 cell migration and induces cell apoptosis by downregulating the expression of COX-2 and VEGF genes. Oncol. Rep..

[21] Gao L., Wang L., Sun Z., Li H., Wang Q., Yi C., Wang X. (2017). Morusin shows potent antitumor activity for human hepatocellular carcinoma in vitro and in vivo through apoptosis induction and angiogenesis inhibition. Drug Des. Devel. Ther..

[22] Li H., Wang Q., Dong L., Liu C., Sun Z., Gao L., Wang X. (2015). Morusin suppresses breast cancer cell growth in vitro and in vivo through C/EBPβ and PPARγ mediated lipoapoptosis. J. Exp. Clin. Cancer Res..

[23] Lim S.L., Park S.Y., Kang S., Park D., Kim S.H., Um J.Y., Jang H.J., Lee J.H., Jeong C.H., Jang J.H. (2014). Morusin induces cell death through inactivating STAT3 signaling in prostate cancer cells. Am. J. Cancer Res..

[24] Liu X.X., Zhang X.W., Wang K., Wang X.Y., Ma W.L., Cao W., Mo D., Sun Y., Li X.Q. (2018). Kuwanon G attenuates atherosclerosis by upregulation of LXRα-ABCA1/ABCG1 and inhibition of NFκB activity in macrophages. Toxicol. Appl. Pharmacol..

[25] Jung H.W., Kang S.Y., Kang J.S., Kim A.R., Woo E.R., Park Y.K. (2014). Effect of Kuwanon G isolated from the root bark of Morus alba on ovalbumin-induced allergic response in a mouse model of asthma. Phytother. Res..

[26] Park K.M., You J.S., Lee H.Y., Baek N.I., Hwang J.K. (2003). Kuwanon G: An antibacterial agent from the root bark of Morus alba against oral pathogens. J. Ethnopharmacol..

[27] Spergel J.M., Paller A.S. (2003). Atopic dermatitis and the atopic march. J. Allergy Clin. Immunol..

[28] Skibola C.F., Smith M.T. (2000). Potential health impacts of excessive flavonoid intake. Free Radic. Biol. Med..

[29] Arellano F.M., Wentworth C.E., Arana A., Fernández C., Paul C.F. (2007). Risk of lymphoma following exposure to calcineurin inhibitors and topical steroids in patients with atopic dermatitis. J. Invest. Dermatol..

[30] Sumiyoshi K., Nakao A., Setoguchi Y., Tsuboi R., Okumura K., Ogawa H. (2003). TGF-beta/Smad signaling inhibits IFN-gamma and TNF-alpha-induced TARC (CCL17) production in HaCaT cells. J. Dermatol. Sci..

[31] Vestergaard C., Bang K., Gesser B., Yoneyama H., Matsushima K., Larsen C.G. (2000). A Th2 chemokine, TARC, produced by keratinocytes may recruit CLA+CCR4+ lymphocytes into lesional atopic dermatitis skin. J. Invest. Dermatol..

[32] Han E.H., Hwang Y.P., Choi J.H., Yang J.H., Seo J.K., Chung Y.C., Jeong H.G. (2011). Psidium guajava extract inhibits thymus and activation-regulated chemokine (TARC/CCL17) production in human keratinocytes by inducing heme oxygenase-1 and blocking NF-κB and STAT1 activation. Environ. Toxicol. Pharmacol..

[33] Kim W.H., An H.J., Kim J.Y., Gwon M.G., Gu H., Lee S.J., Park J.Y., Park K.D., Han S.M., Kim M.K. (2017). Apamin inhibits TNF-α- and IFN-γ-induced inflammatory cytokines and chemokines via suppressions of NF-κB signaling pathway and STAT in human keratinocytes. Pharmacol. Rep..

[34] Ahn S., Siddiqi M.H., Aceituno V.C., Simu S.Y., Zhang J., Perez Z.E., Kim Y.J., Yang D.C. (2016). Ginsenoside Rg5:Rk1 attenuates TNF-α/IFN-γ-induced production of thymus- and activation-regulated chemokine (TARC/CCL17) and LPS-induced NO production via downregulation of NF-κB/p38 MAPK/STAT1 signaling in human keratinocytes and macrophages. In Vitro Cell Dev. Biol. Anim..

[35] Jung M., Lee T.H., Oh H.J., Kim H., Son Y., Lee E.H., Kim J. (2015). Inhibitory effect of 5,6-dihydroergosteol-glucoside on atopic dermatitis-like skin lesions via suppression of NF-κB and STAT activation. J. Dermatol. Sci..

[36] Henderson W.R. (1997). The role of leukotrienes in inflammation. Ann. Intern. Med..

[37] Rezende B.M., Athayde R.M., Gonçalves W.A., Resende C.B., Teles de Tolêdo Bernardes P., Perez D.A., Esper L., Reis A.C., Rachid M.A., Castor M.G.M.E. (2017). Inhibition of 5-lipoxygenase alleviates graft-versus-host disease. J. Exp. Med..

[38] Byrum R.S., Goulet J.L., Griffiths R.J., Koller B.H. (1997). Role of the 5-lipoxygenase-activating protein (FLAP) in murine acute inflammatory responses. J. Exp. Med..

[39] Nedoszytko B., Sokołowska-Wojdyło M., Ruckemann-Dziurdzińska K., Roszkiewicz J., Nowicki R.J. (2014). Chemokines and cytokines network in the pathogenesis of the inflammatory skin diseases: Atopic dermatitis, psoriasis and skin mastocytosis. Postepy. Dermatol. Alergol..

[40] Brandt E.B., Sivaprasad U. (2011). Th2 Cytokines and Atopic Dermatitis. J. Clin. Cell Immunol..

[41] Shefler I., Taube Z., Medalia O., Sagi-Eisenberg R. (1998). Basic secretagogues activate protein tyrosine phosphorylation and release of arachidonic acid in mast cells via a novel protein kinase C and phosphatidylinositol 3-kinase-dependent mechanism. Eur. J. Immunol..

[42] Peng Z., Beaven M.A. (2005). An essential role for phospholipase D in the activation of protein kinase C and degranulation in mast cells. J. Immunol..

[43] Baranes D., Razin E. (1991). Protein kinase C regulates proliferation of mast cells and the expression of the mRNAs of fos and jun proto-oncogenes during activation by IgE-Ag or calcium ionophore A23187. Blood.

[44] Nishida K., Yamasaki S., Ito Y., Kabu K., Hattori K., Tezuka T., Nishizumi H., Kitamura D., Goitsuka R., Geha R.S. (2005). FcɛRI-mediated mast cell degranulation requires calcium-independent microtubule-dependent translocation of granules to the plasma membrane. J. Cell Biol..

[45] Kim O.K., Nam D.E., Lee M., Kwon H.O., Park J., You Y., Kim S.I., Lee J., Jun W. (2016). The Effects of Costaria costata Extracts on Atopic Dermatitis in an In Vitro Model. J. Med. Food.

[46] Lim H.S., Ha H., Lee M.Y., Jin S.E., Jeong S.J., Jeon W.Y., Shin N.R., Sok D.E., Shin H.K. (2014). Saussurea lappa alleviates inflammatory chemokine production in HaCaT cells and house dust mite-induced atopic-like dermatitis in Nc/Nga mice. Food Chem. Toxicol..

